# Impact of Working Memory Load on fMRI Resting State Pattern in Subsequent Resting Phases

**DOI:** 10.1371/journal.pone.0007198

**Published:** 2009-09-25

**Authors:** Martin Pyka, Christian F. Beckmann, Sonja Schöning, Sascha Hauke, Dominik Heider, Harald Kugel, Volker Arolt, Carsten Konrad

**Affiliations:** 1 Department of Psychiatry, University of Münster, Münster, Germany; 2 Interdisciplinary Center for Clinical Research (IZKF FG4), University of Münster, Münster, Germany; 3 Otto Creutzfeld Centre for Cognitive and Behavioral Neuroscience, University of Münster, Münster, Germany; 4 Division of Neuroscience and Mental Health, Imperial College London, London, United Kingdom; 5 Oxford Centre for Functional Magnetic Resonance Imaging of the Brain (FMRIB), University of Oxford, Oxford, United Kingdom; 6 Institute of Computer Science, University of Münster, Münster, Germany; 7 Center for Medical Biotechnology, University of Duisburg-Essen, Essen, Germany; 8 Department of Clinical Radiology, University of Münster, Münster, Germany; 9 Department of Psychiatry and Psychotherapy, University of Marburg, Marburg, Germany; Victoria University of Wellington, New Zealand

## Abstract

**Background:**

The default-mode network (DMN) is a functional network with increasing relevance for psychiatric research, characterized by increased activation at rest and decreased activation during task performance. The degree of DMN deactivation *during* a cognitively demanding task depends on its difficulty. However, the relation of hemodynamic responses in the resting phase *after* a preceding cognitive challenge remains relatively unexplored. We test the hypothesis that the degree of activation of the DMN following cognitive challenge is influenced by the cognitive load of a preceding working-memory task.

**Methodology/Principal Findings:**

Twenty-five healthy subjects were investigated with functional MRI at 3 Tesla while performing a working-memory task with embedded short resting phases. Data were decomposed into statistically independent spatio-temporal components using Tensor Independent Component Analysis (TICA). The DMN was selected using a template-matching procedure. The spatial map contained rest-related activations in the medial frontal cortex, ventral anterior and posterior cingulate cortex. The time course of the DMN revealed increased activation at rest after 1-back and 2-back blocks compared to the activation after a 0-back block.

**Conclusion/Significance:**

We present evidence that a cognitively challenging working-memory task is followed by greater activation of the DMN than a simple letter-matching task. This might be interpreted as a functional correlate of self-evaluation and reflection of the preceding task or as relocation of cerebral resources representing recovery from high cognitive demands. This finding is highly relevant for neuroimaging studies which include resting phases in cognitive tasks as stable baseline conditions. Further studies investigating the DMN should take possible interactions of tasks and subsequent resting phases into account.

## Introduction

Resting-state networks (RSN) have recently attracted increased attention in neuroscience research. In contrast to task-related increases of brain activation in functional networks, usually the focus of neuroimaging research, RSNs have been characterized as functionally distinct networks that represent the baseline activity of the human brain in the absence of stimulated neuronal activity (e.g. [1]–[5]). RSNs also seem to be related to coherent oscillations in widely separated brain regions [2], [6]–[9]. Reports about functional networks denoted as RSNs have been reproduced several times using different numerical approaches, such as correlation analysis [10]–[12] and independent component analysis (ICA) [7], [13]–[15].

One prominent RSN is the so-called default-mode network (DMN) that comprises medial frontal regions, the ventral anterior cingulate cortex (vACC), precuneus, the posterior cingulate cortex (PCC) and the lateral parietal cortex [3], [13], [16]. The DMN consistently occurs in the unstimulated (resting) brain [1], [7], [9], [12], [13], [17], and is significantly more activated during rest, passive sensory and visual processing than during cognitively demanding tasks, when goal-directed behavior is involved [4], [16], [18]–[23]. Furthermore, the magnitude of the deactivation increases with task difficulty [20]–[23] accompanied with a reduced frequency of task-unrelated thoughts [24]. These observations underline the notion that DMN deactivations reflect relocation of cerebral resources in order to support task performance [25], [26]. Conversely, increased activation of the network has been suggested to be associated with processes of mind wandering [16], readiness [9] and self-referential thoughts [3]. In some brain areas, including the medial and lateral frontal and medial parietal cortex, task-induced decreases are inversely correlated with improvement of task performance in the course of motor sequence learning [14]. Furthermore, studies revealed age-dependent effects on the DMN. In younger subjects the decrease of activation in the DMN during a working-memory task is more pronounced than in older subjects [27]–[29]. However, a greater deactivation of the left ACC in older adults has been observed during the retrieval of paired associates [21]. Recently, the DMN has also gained high importance in the investigation of psychiatric diseases (for review, see [30]), such as major depression [31], [32], Alzheimer disease [21], [27], [33], [34] or schizophrenia [35], [36].

While many studies investigated the activation of the DMN during a challenging task, much less is known about the potential impact of a cognitive task on the subsequent resting phase. A functional connectivity analysis of specific DMN regions in resting phases before and after an orthographic lexical retrieval task revealed relatively small increases and decreases in functional connectivity between both resting sessions [37]. But given that the DMN is known to be a modulator of self-referential processes, we hypothesize that cognitively demanding working-memory processes might exert an influence on the magnitude of activation of the DMN in the subsequent resting phase, e.g. as a neuronal correlate of subsequent self-evaluation and reflection. Therefore, we expect that the activation of the DMN at rest is greater after a cognitively challenging task than after a task with low mental effort. We applied Independent Component Analysis (ICA), a well-established approach for detecting underlying networks [38], [39], to analyze the interaction between the cognitive load of a working-memory task and the subsequent activity of the DMN in short resting phases (24 s) between task blocks. In contrast to Greicius et al. [18], [39] who investigated the DMN in a working-memory and a resting condition, the resting phases here are embedded in the working-memory paradigm in order to analyze possible interactions between load of the working-memory task and the subsequent resting phase. Unlike Waites et al. [37], block and rest phase lasted only 36 and 24 seconds (instead of 5:24 min for each block), and were repeated twice, allowing a more sensitive analysis of short-term effects of the task. We addressed the following questions: i. Can the modulatory effect of cognitive load on the deactivation of the DMN *during* task performance be confirmed? ii. Does the load of a working-memory task exert an influence on the activation of the DMN in the *subsequent* resting phase?

## Methods

### Subjects

Twenty-five healthy right-handed volunteers participated in this study (mean age: 32.4 years, range: 18–55; 13 female, 12 male; mean IQ: 119.2). An initial telephone screening was conducted to ensure inclusion criteria, to exclude medical and neurological diseases or MRI contraindications. The SCID-I-interview was performed to exclude any current or previous psychiatric disorders [40]. Furthermore, inclusion criteria involved no psychiatric disorders in first degree relatives and a minimum of twelve years of education. All procedures were approved by the Institutional Ethical Review Board of the medical association Westfalen-Lippe and the medical faculty of the University of Münster. The ethical standards of the Declaration of Helsinki were met and all participants provided written informed consent.

### Material and procedures

The working-memory task was part of a larger fMRI and neuropsychological study of memory processes [41]. We used a classical letter variant of the n-back task [42] with a resting block after each n-back task. The letter sequence was presented in a block design consisting of 12 consonants, including one third targets. For the 0-back condition subjects had to decide whether the target letter “X” appeared on the screen. In the 1-back condition, the target letters were defined as those letters that appeared in the previous trial. During 2-back, subjects had to decide whether the actual letter was identical to the letter presented two trials before. Subjects responded with the right hand, using the index finger for targets and middle finger for non-targets.

Each n-back block (0-back, 1-back, 2-back) lasted 36 s, each subsequent rest block 21 s plus 3 s instruction for the next task (R0-back, R1-back, R2-back). N-back blocks and rest blocks were presented in a fixed order (1-back R1-back, 0-back R0-back, 2-back R2-back, 0-back R0-back, 1-back R1-back, 2-back R2-back). During the resting phase, participants looked at a white fixation cross on a black screen. Subjects were instructed to relax and wait until the next n-back-block starts. Letters were presented in white in the centre of a black screen for 500 ms and an interstimulus interval of 2500 ms (Presentation Software®, Version 10.81, 2004, Neurobehavioral Systems Inc., Albany, CA, USA).

### Scanning procedures

Functional images were acquired on a 3 Tesla whole-body scanner (Intera T 3.0, Philips, Best, NL). We used a circularly polarized transmit/receive birdcage head coil with an HF reflecting screen at the cranial end for spin excitation and resonance signal acquisition. One hundred and twenty whole-brain T2* weighted single shot echo planar (EPI) images were acquired (sequence parameters: TE = 38 ms, TR = 3000 ms, flip angle 90°, slice thickness 3.6 mm without gap, matrix 64×64, FOV 230 mm, in-plane resolution 3.6×3.6 mm2). Thirty-six transversal slices orientated to the AC-PC line were acquired.

### Behavioral data analysis

Responses and response latencies (in ms) for the n-back performance were recorded during fMRI scanning. Performance is reported as accuracy rate (percentage of correct answers) for each n-back condition. We performed repeated-measures analyses of variance (ANOVAs) with one within-subject factor (working-memory load: three levels) for accuracy rate and response latency.

### Functional data analysis

Data were pre-processed using tools from the FMRIB Software Library (FSL, http://www.fmrib.ox.ac.uk/fsl) [43]. The following procedures were applied: motion correction using MCFLIRT, spatial smoothing using a Gaussian kernel of FWHM 5 mm, mean-based intensity normalization of all volumes by the global factor and high-pass temporal filtering.

Following pre-processing, data were analyzed using Tensor-ICA (TICA) [38] provided in MELODIC (Multivariate Exploratory Linear Optimized Decomposition into Independent Components) as part of the FSL. TICA is a derivative of Parallel Factor Analysis (PARAFAC, [44]) which separates the multi-subject data into sets of vectors characterizing underlying signals in the temporal, spatial and subject domain. While probabilistic ICA (PICA) is appropriate for resting-fMRI and multi-subject data with non-uniform time-courses associated with each underlying signal, we here employed a full tensorial ICA approach in order to extract subject/session specific differences in multi-subject data (assuming a consistent task-related paradigm in all subject data). MELODIC determined the number of independent components using a Laplace approximation to the Bayesian evidence of the model order, resulting in a 31-dimensional signal subspace (see [38] for details). These 31 independent components were calculated by projecting the subject's data into the space of the 31 eigenvectors and optimizing for spatial independence between component maps using a measure of non-Gaussianity as the ICA cost function [45]. Final spatial components were normalized to z-scores reflecting the degree to which a given voxel's time-series correlates with the overall component time-series (i.e. scaled by the standard deviation of the residual Gaussian noise)(see [46] for details).

To select a component corresponding to the DMN, components with motion related artifacts or with a mean power above 0.1 Hz were excluded from further analysis. Furthermore, since TICA is able to extract the subject-/session-domain specific effect magnitude for each spatio-temporal functional activation pattern, we also excluded all components driven by a single outlier subject. For the detection of a rest-related component we used an approach described by [47]. Z-scores of all 31 components were compared with a predefined DMN template as provided by Greicius et al. [33] in order to detect the component with the “best fit”. This template is a statistical map of a second-level random effects analysis in which 14 healthy subjects conducted a resting-state study. As suggested by Greicius and Menon [47], the average z-scores of the voxels situated within the template were subtracted by the average z-score of the voxels outside the template, allowing determination of the component with the best goodness-of-fit. To assure that solely the selected component matched the template, a two-sample t-test was calculated for the best-fit and the second best-fit component.

A repeated measures ANOVA with two within-subject factors LOAD (0-, 1-, 2-back: 3 levels) and PHASE (activation, rest: 2 levels) was calculated for the dependant variable DMN activation with six repetitions (mean values of each phase 0-back, 1-back, 2-back, R0-back, R1-back, and R2-back), enabling the investigation of interaction effects of LOAD and PHASE in the resting state component. Post-hoc tests were conducted to test specifically the hypothesis that resting activity after a cognitive demanding working-memory task (e.g. 1-back, 2-back) has a greater magnitude compared to resting activity after 0-back. Since other studies reported that inter-subject variability of DMN activation can be fairly large [24], [37], we choose Fisher LSD for post-hoc tests and subsequent inferences, as it accounts for a more sensitive testing of mean value differences.

## Results

### Behavioral Results

**Figure 1:**
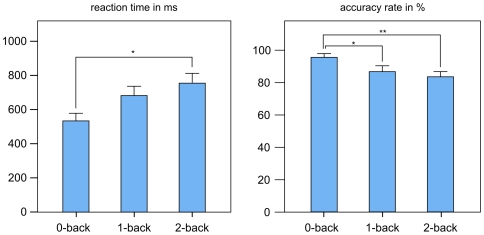
Reaction times and accuracy rate. Reaction times (mean±standard error) and accuracy rate (percentage of correct answers, mean±standard error) of 25 subjects. Asterisks denote where pair-wise comparison is significant.

Analyses of variance of accuracy and on response latency showed a significant main effect of working-memory load (F(2,48) = 8.75, p<0.001) and (F(2,48) = 17.36, p<0.001), respectively. With increasing working-memory load, accuracy decreased and response latency increased (Figure 1). Post-hoc tests (Fisher LSD) revealed pair-wise significant differences between 0-back and 2-back in reaction times and accuracy rate (p = 0.005, p = 0.003) and 0-back and 1-back in accuracy rate (p = 0.025). Further information with regard to the parametric effects of working memory load in this experiment have been reported in [41].

### Evaluation of TICA components

TICA was applied to 25 healthy subjects. The temporal domain of the data was reduced to 31 independent components explaining 80% of the total variance. Fifteen components did not meet the criteria for RSN maps described in the methods section. Ten of them contained motion related artefacts, three components were solely present in one subject, and two more had a frequency spectrum of more than 50% above 0.1 Hz. For the remaining components, the described template-matching procedure was applied to find the best-fit component of the default mode network.

### Goodness-of-Fit Scores

The goodness-of-fit score for the best-fit component was 4.13 and for the second best-fit component 1.60. A two-sample t-test showed that both components differ significantly (p<0.001). Note, that a goodness-of-fit score represents the average z-score of voxels falling within the template minus the average z-score of voxels falling outside the template. Hence, the largeness of the value depends on the range of the z-scores.

### Time course of the default-mode network

**Figure 2:**
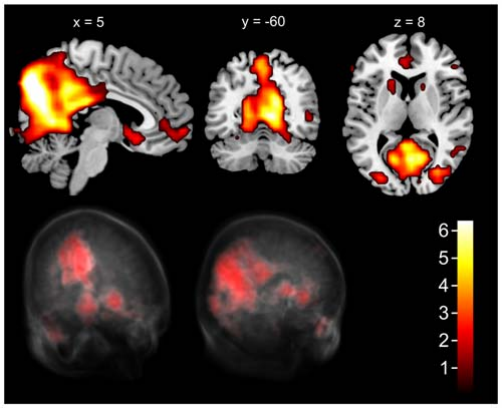
Default-mode network extracted by TICA. Top: sagittal, coronal and transversal view (Image displayed in radiological convention). Bottom: rendered 3d-view with transparent voxeldata from frontal-left- (left) and behind-left-view (right). Major clusters with coordinates, Z-score and Brodman area (BA) are: PCC (−4, −32, 28), Z = 5.86, BA 31 and (−6, −52, 12), Z = 3.87, BA 30; Precuneus (−8, −72, 28), Z = 6.44, BA 31; Cuneus (10, −74, 30), Z = 7.50, BA 18; Culmen (−8, −48, −4), Z = 3.09, BA 19; vACC (4, 16, −12), Z = 2.86, BA 25; Middle Frontal Gyrus (4, 60, −4), Z = 3.33, BA 10. Clusters exist left and right in similar size.

**Figure 3:**
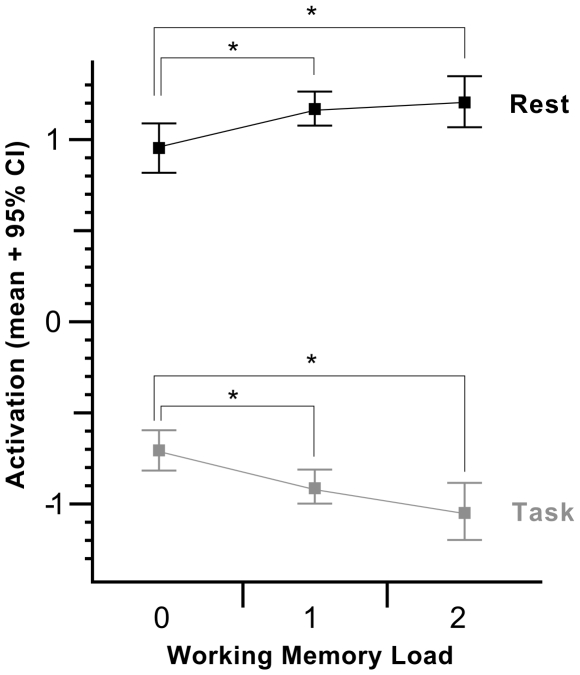
Mean of activation in the DMN. Mean of activation in the DMN were calculated for each n-back (lower values: 0-back, 1-back, 2-back) and subsequent resting block (upper values: R0-back, R1-back, R2-back). x-Axis: working memory load, y-Axis: activation as extracted by TICA for the selected component (which can differ in scaling and mean).

The selected resting-related component showed activations in the vACC and in posterior parietal and medial occipital areas (Figure 2). The corresponding time course was strongly negatively correlated with the task-rest-sequence (Pearson's r = −0.91; p<0.0001), that is the activation of the network decreased during an n-back block and increased during rest phases. The areas are also included in RSN 2 given in [7] and are similar to those areas described by [47]. Based on the paradigm the overall mean of activation for each LOAD/PHASE combination was calculated for each subject. A repeated-measures ANOVA for the within-subject factors load (0-back, 1-back, 2-back) and phase (activation, rest) revealed that the mean of activation respectively deactivation is significantly modulated by phase (F(1, 24)>1000; p<0.001) and the interaction between load and phase alone (F(2, 48) = 10.24; p = 0.01). No significant influence could be found for the factor load (F(2, 48) = 0.56; p = 0.58). Post-hoc tests by Fischer LSD confirmed that not only deactivation between different n-back-tasks is significantly different but also activation of the DMN in rest differs depending on the preceding n-back-task (Table 1). Figure 3 illustrates the mean of activation for each load and rest condition.

**Table 1:** Main effects and Fischer LSD Post-hoc tests for mean activation changes during working memory task and subsequent rest.

**Anova**	**F**	**p**
Load	F(2, 48) = 0.56	0.58
Phase	F(1, 24)>1000	<0.001***
Load*Phase	F(2, 48) = 10.24	0.01*
**Post-Hoc Tests**		**p**
0-back > Activation 1-back		0.002**
0-back > Activation 2-back		<0.001***
1-back > Activation 2-back		>0.05
Rest 1-back > Rest 0-back		0.037*
Rest 2-back > Rest 0-back		0.041*
Rest 2-back > Rest 1-back		>0.05

## Discussion

This study applied TICA to 25 healthy subjects to investigate the influence of cognitive load in a working-memory task on subsequent resting phases. We determined the DMN using an unbiased template matching procedure [18]. This component contains the vACC, a medial frontal area, the PCC and medial parietal areas. Consistent with previous studies, we found that the DMN is stronger deactivated with increased cognitive demands during the working-memory task. Furthermore, we also provide evidence that the activation of the DMN is more pronounced after a cognitively challenging task (1-back, 2-back) compared to a simple letter detection task (0-back) (Figure 3).

From a methodological perspective, a model-free approach such as TICA is an efficient method for identifying time courses of activation of such functional networks. Unlike the analysis based on the general linear model (GLM), the definition of a stable baseline is not necessary. TICA enables the investigation of resting phases between cognitive tasks, which in the GLM are usually considered to correspond to a stable baseline. Since the time-course of the DMN, extracted by ICA, does not represent any relative differences to a certain condition, the increased resting-state activation does apparently not just reflect the negative difference between a baseline and a demanding task. Therefore this approach may reveal task-related shifts in embedded rest phases. The use of TICA is therefore warranted even in designs when the GLM might also be applicable.

As a first result of our study, we confirm that deactivation of the DMN during task performance increases with task difficulty, which is in line with other studies investigating the DMN in working-memory paradigms (e.g. [21]–[23]). McKiernan and colleagues manipulated cognitive load using an auditory working-memory task and detected an increasing magnitude of deactivation in the DMN with increasing task difficulty [23]. This result indicates that cognitive resources are reallocated according to the demands of cognitive tasks. The performance of cognitively demanding tasks therefore results in deactivation of the DMN. Similar findings have been reported for the DMN and the medial temporal lobe whose magnitude of deactivation (compared to periods of rest) correlates with superior memory ability [48]. With performance enhancement, the need for relocation of resources decreases. In a motor sequence learning study, Tamás Kincses et al. [14] demonstrated that some of the rest-related components correlate significantly with behavioral improvement during the course of learning. It is still part of an ongoing debate how the reported behavior of the DMN in response to exogenous stimuli can be interpreted [3], [5], [18], [22], [49]. One common view is that DMN activity reflects a functional correlate of internal generated thought processes that are interfered by perceptual and short-term memory demands [3], [18], [23], [24]. The relation between these internally and externally generated signals have been the focus of numerous laboratory studies in the past decades [50]–[54]. As the degree of interference seems to depend on the processing demands for external stimuli, it has been suggested that endogenous and exogenous signals compete for processing resources. In this regard, studies that reported a correlation between task-related deactivation of the DMN and lower frequency of task-unrelated thoughts [16], [24], might reflect a functional representation of this idea. The broad range of exogenous stimuli that cause deactivation in DMN related areas lead to the view that endogenous processes need to be inhibited for the successful execution of a task [22]. This suppression of internal mental activity are thought to enable the reallocation of attentional resources from internal thought processes to goal-directed behavior [21].

As the main result of this study, we showed that the DMN was more active in the rest phase after 2-back and 1-back than during rest after 0-back. We hypothesize that DMN activation is influenced by cognitive load not only during but also after cognitive challenging tasks. Previous studies investigating rest phases after cognitive load specifically focused on demonstrating the stability of the DMN and regional connectivity over time. Waites et al. [37] investigated rest phases before and after an orthographic lexical retrieval task by comparing functional connectivity maps of certain seed regions, including the PCC. The group analysis revealed only very subtle differences in connectivity between both resting-state sessions. These results show that the time course of the selected independent component, extracted by TICA, reflects the activation of a spatially robust functional network. However, in this study the load of the cognitive task was not manipulated. Likewise, Hampson et al. confirmed that functional connectivity between the PCC and a region in the medial frontal gyrus/vACC is preserved at rest and during a working-memory task [55]. Working-memory performance was positively correlated with the strength of the functional connectivity between those two areas, even in subsequent rest phases. None of these studies, however, investigated the influence of a variable cognitive load on the magnitude of DMN activation in subsequent rest phases.

Conclusions regarding the psychological role of the DMN can only be derived indirectly from our data, as the conducted experiment did not include reports of the subjects' mental processes after the n-back blocks. The increased activation of the DMN after 1- and 2-back could be interpreted as increased redistribution of cognitive resources in the subsequent rest phase, reflecting a neuronal correlate of mental fatigue. Previous studies investigated the functional correlates of mental fatigue with respect to chronic diseases, such as fatigue syndrome, but focused only on task-related brain areas or on differences between patients and controls [56], [57]. Therefore, the neuro-functional impact of task-induced temporal fatigue on subsequent rest remains unexplored so far. But since task and rest phases lasted only 36 and 24 seconds, it is questionable whether the presented n-back blocks were long enough to induce cognitive fatigue. Additionally, we would expect a stronger amplitude after the 2-back block. With respect to previous publications, the DMN has been implicated in processes of mind-wandering [16], but also retrieval and manipulation of past events [18]. Studies addressing self-referential processes in cortical midline structures reveal that processes of reflection and judgment on personality traits are associated with typical DMN areas (for review, see [58]). Thus we hypothesize that increased cognitive effort during a task-block might lead to stronger subsequent processes of self-evaluation and reflection of preceding events resulting in increased activation of the DMN. In this context, activity of the DMN would not only reflect the degree of suppression of the “stream of consciousness” [24], but also the responsiveness to which cognitive tasks leads to subsequent processes of self-referential thoughts.

While the n-back task increases working-memory effort parametrically, the decrease and increase of DMN activation seems to be attenuated from 1-back to 2-back. The observed non-parametric decrease during the task condition corresponds to previous findings [19], [22], [23]. McKiernan et al. [23], explained the non-parametric nature of the decrease during cognitive task performance by a “ceiling effect” for the moderate and difficult condition. In our experiment, the nonparametric decrease during working-memory performance may also be explained by the fact that redistribution of resources with decreased attention to free internal thought processing [4] is already high during performance of the 1-back task. Concerning the nonlinear DMN increase after the n-back task, which probably reflects increased self-referential processes analogous to [9], [24], it appears plausible that DMN activity does not necessarily follow a linear course. As self-referential processes might already be activated by minor increases in preceding task complexity, they do not directly reflect the previous task-related magnitude of reallocation of cerebral resources. Furthermore, while parametric increases of working-memory demands occur in areas of the working-memory network [41], the reallocation of cognitive resources occurs in many other, distinct brain areas including the DMN network. It seems plausible that the reallocation of resources therefore follows a nonlinear function in brain areas which differ from the working-memory network itself.

The functional role of the DMN has been described in the literature with concepts, such as task-unrelated thoughts, stream of consciousness, self-referential processes, verbal and visual imagery, planning and problem solving, internal monitoring and episodic memory encoding [3], [4], [5], [26], [49], [59]. Although we hypothesize, that the reported effect might be related to increased processes of self-reflection after a challenging task, the findings probably also reflect several, overlapping cognitive processes that have been associated with the DMN and cannot easily be separated from each other. Thus, further studies are necessary to discriminate between different cognitive processes that are involved in DMN activation and deactivation. Multi-modal experimental tasks (as used in [14]) as well as self-reports of subjects (see [24] and [16]) might help to clarify the relation between different mental processes and their neuro-functional correlate.

Some limitations need to be considered. Although the sample size is high for a functional imaging study and inclusion criteria were rigorous, we cannot completely exclude that our results are influenced by the selection of the cohort. Moreover, the complex behavior of the DMN and the inter-subject variability that has been noticed by us and other groups weaken the results. Another limitation might be the short resting phase between the n-back blocks which does not allow the investigation of the temporal dynamics of the DMN deactivation during a longer period of time. Future studies should integrate a longer resting period in their working-memory task to examine this issue. However, the short resting phase of 24 s in our study underlines the intensity of the described interaction effect demonstrating the modulation of baseline activity by preceding cognitive tasks. Furthermore, we suggest that future investigations should assess psychological variables about self-referential thoughts during the rest phases.

In summary, we demonstrated that cognitively challenging working-memory tasks have a significant influence on the activation of the DMN in subsequent resting phases. Thus, we provide evidence that, even at rest, functional processes are affected by the preceding cognitive task. In the framework of a GLM, the reported baseline shifts might lead to overestimated brain activation or deactivation during task performance if they are not included as regressors in the classical multivariate analysis. How this effect is related to self-referential processes in response to a preceding task needs to be investigated in future studies.
